# Dominant and recessive mutations in the Raf-like kinase *HT1* gene completely disrupt stomatal responses to CO_2_ in Arabidopsis

**DOI:** 10.1093/jxb/erw134

**Published:** 2016-03-31

**Authors:** Mimi Hashimoto-Sugimoto, Juntaro Negi, Keina Monda, Takumi Higaki, Yasuhiro Isogai, Toshiaki Nakano, Seiichiro Hasezawa, Koh Iba

**Affiliations:** ^1^Department of Biology, Faculty of Science, Kyushu University, Fukuoka 819-0395, Japan.; ^2^Department of Integrated Biosciences, Graduate School of Frontier Sciences, The University of Tokyo, Kashiwanoha Kashiwa, Chiba 277–8562, Japan.; ^3^Department of Biotechnology, Faculty of Engineering, Biotechnology Research Center, Toyama Prefectural University, 5180 Kurokawa, Imizu, Toyama 939-0398, Japan.

**Keywords:** ABA, Arabidopsis, CO2 response, HIGH LEAF TEMPERATURE 1, Light, MAPKKK, Raf-like kinase, stomatal control.

## Abstract

HT1 (HIGH LEAF TEMPERATURE 1) is the first component associated with changes in stomatal aperture in response to CO_2_ to be isolated by forward genetic screening. The *HT1* gene encodes a protein kinase expressed mainly in guard cells. The loss-of-function *ht1-1* and *ht1-2* mutants in *Arabidopsis thaliana* have CO_2_-hypersensitive stomatal closure with concomitant reductions in their kinase activities *in vitro*. In addition to these mutants, in this study we isolate or obtaine five new *ht1* alleles (*ht1-3*, *ht1-4*, *ht1-5*, *ht1-6*, and *ht1-7*). Among the mutants, only *ht1-3* has a dominant mutant phenotype and has widely opened stomata due to CO_2_ insensitivity. The *ht1-3* mutant has a missense mutation affecting a non-conserved residue (R102K), whereas the other six recessive mutants have mutations in highly conserved residues in the catalytic domains required for kinase activity. We found that the dominant mutation does not affect the expression of *HT1* or the ability to phosphorylate casein, a universal kinase substrate, but it does affect autophosphorylation activity *in vitro*. A 3D structural model of HT1 also shows that the R102 residue protrudes from the surface of the kinase, implying a role for the formation of oligomers and/or interaction with its targets. We demonstrate that both the loss-of-function and gain-of-function *ht1* mutants have completely disrupted CO_2_ responses, although they have normal responses to ABA. Furthermore, light-induced stomatal opening is smaller in *ht1-3* and much smaller in *ht1-2*. Taken together, these results indicate that HT1 is a critical regulator for CO_2_ signaling and is partially involved in the light-induced stomatal opening pathway.

## Highlight

Loss-of-function and gain-of-function *ht1* Arabidopsis mutants have completely disrupted CO_2_ responses due to reduced and enhanced kinase activities, respectively.

## Introduction

Plants control CO_2_ uptake for photosynthesis and regulate transpirational water loss through stomatal pores. The two guard cells forming each pore are able to sense environmental signals and endogenous stimuli, integrate this information and optimize stomatal aperture size (Hetherington and Woodward, 2003; Shimazaki *et al.*, 2007; Kim *et al.*, 2010; Kollist *et al.*, 2014). Stomata open in response to low CO_2_ concentration ([CO_2_]) to prevent a decrease in CO_2_ uptake and close at high [CO_2_] in order to maintain high values of water-use efficiency. Stomata are thought to respond to the intercellular [CO_2_] (*C*_i_) changes caused by photosynthesis and transpiration (Mott, 1988; Assmann, 1999). The global annual mean concentration of atmospheric CO_2_ has steadily increased from a pre-industrial value of about 280ppm to 395ppm as of 2013 (National Oceanic and Atmospheric Administration data; http://www.esrl.noaa.gov/gmd/ccgg/insitu.html). This rise in [CO_2_] has caused a significant decrease in stomatal conductance in many species on a global scale (Medlyn *et al.*, 2001) and affects plant ecosystems (Ainsworth and Rogers, 2007; Keenan *et al.*, 2013). Understanding the molecular basis for stomatal sensitivity to atmospheric CO_2_ will improve our ability to predict future ecosystem responses; however, the detailed mechanisms by which CO_2_ effects changes in stomatal aperture remain largely unknown.

Stomatal pore size is regulated by the turgor pressure of the guard cells. Stomatal opening is driven by an increase in guard-cell turgor when plasma membrane H^+^-ATPases are activated (Shimazaki *et al.*, 2007). The activated H^+^-ATPases induce membrane hyperpolarization, thereby facilitating K^+^ entry, which in turn causes solute influx followed by water uptake into the guard cells (Schroeder *et al.*, 1984). Elevated [CO_2_] has been suggested to inhibit proton pumps (Edwards and Bowling, 1985) and activate anion channels and K^+^_out_ efflux channels in the guard cells (Brearley *et al.*, 1997; Roelfsema *et al.*, 2002; Raschke *et al.*, 2003). These changes result in chloride release from the guard cells, membrane depolarization, the loss of guard-cell turgor, and thus stomatal closure (Hanstein and Felle, 2002).

Recently, mutant screening and functional characterization have led to the identification of plant mutants and genes involved in CO_2_ signaling (Israelsson *et al.*, 2006; Negi *et al.*, 2014). The first *Arabidopsis thaliana* mutant with a defective stomatal CO_2_ response, *ht1* (*high leaf temperature 1*), was isolated by analysing leaf temperature changes using thermography (Hashimoto *et al.*, 2006). *HT1* encodes a protein kinase mainly expressed in the guard cells, and the two allelic mutations, *ht1-1* and *ht1-2*, cause reduced and no kinase activity, respectively. These altered activities are correlated with unusual stomatal CO_2_ responses: stomatal opening in response to low [CO_2_] is impaired in both mutants; the *ht1-1* mutant has a reduced CO_2_ response; and the *ht1-2* mutant has a severely impaired CO_2_ response leading to constitutively high-[CO_2_] induced stomatal closure. In Arabidopsis, disruption of two carbonic anhydrases, βCA1 and βCA4, also leads to reduced changes in stomatal aperture in response to [CO_2_] changes (Hu *et al.*, 2010). The triple mutant *ca1ca4ht1-2* has an impaired response to CO_2_ similar to that of *ht1-2*, indicating that HT1 is epistatic to the genes for these carbonic anhydrases (Hu *et al.*, 2010). Elevated intracellular bicarbonate and CO_2_ levels activate S-type anion channels including SLAC1 in the guard cells (Hu *et al.*, 2010; Xue *et al.*, 2011). The SLAC1 anion channel is required for ABA- and CO_2_-induced stomatal closure (Negi *et al.*, 2008; Vahisalu *et al.*, 2008). The OST1 protein kinase has been isolated as an ABA-signaling regulator and shown to activate SLAC1 anion channels (Mustilli *et al.*, 2002; Geiger *et al.*, 2009; Lee *et al.*, 2009). Recent findings have shown that the loss-of-function alleles of *OST1* are impaired in the HCO_3_^−^ activation of anion channels, suggesting that OST1 is an ABA and CO_2_ signaling component (Xue *et al.*, 2011). Tian *et al.* (2015) reported that a MATE-type transporter, RHC1, is activated by bicarbonate and functions upstream of HT1. Furthermore, HT1 directly phosphorylates OST1 and inhibits OST1-induced activation of SLAC1 (Tian *et al.*, 2015).

In this study, we demonstrate that not only loss-of-function but also gain-of-function *ht1* mutations completely disrupt CO_2_-regulated stomatal aperture changes. Collectively, these mutants are the most severely compromised phenotypes for CO_2_-signaling among the mutants reported to date. This finding indicates that CO_2_ signaling pathways associated with HT1 have not been completely explained yet.

## Materials and methods

### Plant material and growth conditions

The *Arabidopsis thaliana* wild type (WT) accessions used in this study were derived from the Columbia (Col-0) background unless otherwise noted. EMS-mutagenized Col M_2_ seeds were purchased from Lehle Seeds (Round Rock, TX, USA). We obtained *ht1-6* [stock number CS93263, Col *erecta* (Col *er*) background] from the Arabidopsis TILLING project (http://tilling.fhcrc.org) and the *ht1-7* T-DNA insertional mutant line [FLAG_446H04, Wassilewskija (Ws) background] from the Versailles Arabidopsis Stock Center (http://dbsgap.versailles.inra.fr/publiclines/). Arabidopsis seeds were surface-sterilized and grown on solid 1/2 MS medium for 18 d in a growth chamber [constant white light of 80 µmol m^−2^ s^−1^ at 22 °C, 60% relative humidity (RH)]. The plants were then transplanted into pots with vermiculite and grown for 3 d. These 3-week-old plants were then used for experiments unless otherwise noted.

### Thermal imaging

Thermal imaging of plants was performed as described previously (Hashimoto *et al.*, 2006). The 3-week-old plants were transferred to a growth cabinet (constant white light of 40 µmol m^−2^ s^−1^ at 22 ^o^C, 40% RH) equipped with an automatic CO_2_ control unit (FR-SP, Koito). Thermal images of plants were captured under different [CO_2_] conditions using a thermography apparatus (TVS-8500, Nippon Avionics). Images were fed into a Windows-based computer and were analysed using the software GTStudio (Nippon Avionics). The image processing program Image J (http://imagej.nih.gov/ij/) was also used to quantify leaf temperatures. Details of replication are given in the figure captions.

### Stomatal conductance

Gas exchange was measured on the aerial tissues of 24-d-old seedlings using a portable gas exchange system (GFS3000, Heinz Walz, Effeltrich, Germany) equipped with a 3010-A Arabidopsis chamber (Monda *et al.*, 2011). The GFS3000 system was connected to a computer equipped with data acquisition software (GFS-Win). The cuvette for Arabidopsis conditions was set at a light intensity of 200 µmol m^−2^ s^−1^, provided by a special artificial light (LED-Array/PAMFluorometer 3055-Fl, Heinz Walz, Effeltrich, Germany), with relative humidity and air temperature being set at 50% and 22 ^o^C, respectively. All measurements were made every 60s. Details of replication are given in the figure captions.

### Stomatal aperture response analyses

Stomatal aperture measurements were performed as described previously (Hashimoto *et al.*, 2006). Three-week-old plants were incubated at the selected [CO_2_] in a growth cabinet. Abaxial epidermal peels of the plants were taken from the sixth or seventh leaf and were used immediately to measure stomatal apertures. Leaves from 4- to 5-week-old plants were floated on solutions containing 30mM KCl, 1mM CaCl_2_ and 5mM MES-KOH, pH 6.15, and were incubated in a growth chamber. ABA from a stock solution in dimethyl sulfoxide (DMSO) was added to the solution after 2h of illumination, and stomatal apertures were measured 2h later from epidermal peels using a digital camera attached to a microscope (BH2, OLYMPUS, Tokyo Japan).

### Quantitative reverse transcription PCR (qRT-PCR)

Total RNA was extracted with TRIzol reagent (Invitrogen) according to the manufacturer’s protocol. cDNAs synthesized from total RNA were used as qRT-PCR templates according to a previously described method (Hashimoto *et al.*, 2006). Quantitative PCR was performed as described previously (Negi *et al.*, 2013). Gene-specific signals were normalized relative to *Arabidopsis UBQ10* expression. The primers used in the qRT-PCR analyses were as follows: *HT1*, 5′-GGGCTAAGCTTGAACAACAGT-3′ and 5′-GCGAGTAAGGCTCTTTCTTG -3′; *UBQ10*, 5′-GGCCTTGTATAATCCCTGATGAATAAG-3′ and 5′-AAAGAGATAACAGGAACGGAAACATAGT-3′.

### Preparation of recombinant proteins

The His-tagged recombinant HT1 (His-HT1) and the HT1 protein with the *ht1-3* mutation (His-HT1^R102K^) were expressed and purified from *E. coli* as described previously (Hashimoto *et al.*, 2006). *NdeI* sites were introduced in front of the ATG start codon of *HT1* and *HT1* with the *ht1-3* mutation by PCR using each cDNA as a template. The constructs were then ligated in-frame into the pET-28a (+) vector (Novagen) and were confirmed by DNA sequencing. BL21(DE3) cells transformed with pET-28a (+) constructs were induced with 1mM IPTG for 16h at 25 °C. His-tagged proteins were purified on nickel columns (Amersham Biosciences). Purified His-tagged proteins were recognized specifically by anti-His-probe antibodies (Toyobo) in an immunoblot analysis.

### *In vitro* phosphorylation assay

The kinase assay was performed as described previously (Hashimoto *et al.*, 2006). For the His-HT1 or His-HT1^R102K^ kinase assay, purified recombinant proteins (1 µg) were incubated in a reaction buffer (25mM Tris, pH 7.5, 10mM MgCl_2_) with 1mM CaCl_2_ or calcium chelators (1mM EGTA, 20 µM BAPTA) in the presence of 0.6 µCi [γ-^32^P]ATP at 30 °C for 15min. The reaction was also performed with 100 µM kinase inhibitors (GW5074, ML-9, K252a, staurosporine and genistein) in the reaction buffer. A negative control containing 20 units CIAP (*c*alf *i*ntestinal alkaline *p*hosphatase) was also used in this assay. These reactions were stopped by the addition of SDS-loading buffer, and the proteins were resolved on a 12% SDS-polyacrylamide gel. The proteins were visualized by Coomassie staining with InstantBlue (Ex-pedeon) to verify equal loading, and the kinase activities were detected by autoradiography. Phosphorylation activities of HT1 and its mutants were determined in 10 µl of the kinase reaction buffer using 0.15 µg casein as a substrate under the same reaction conditions. ImageJ software was used to quantify gel bands from the SDS-polyacrylamide gels and the kinase assays.

### Transgenic plants

The *HT1* genomic region (nucleotides 54586 to 58950 of BAC F24O1) containing At1g62400 was amplified by PCR from the genomic DNA of the *ht1-3* mutant using the oligonucleotide primers 5′-CTTCTCTAAGCTTTCGATGCAAACCA-3′ and 5′- GATGTATTGCAAGAGCTGATCAATTGGGTCATGAGA CGAC-3′ and was then inserted into the pGEM-T Easy Vector (Promega). A SalI-MunI fragment including the *HT1* genomic sequences with the *ht1-3* mutation was cloned into the SalI/EcoRI site of the T-DNA vector pBI101. For 35S:*GFP-HT1*, a modified *GFP* ORF fragment with a glycine linker obtained by PCR using primers 5′- ACCATGGTGAGCAAGGGCGA-3′ and 5′- ACATATGAGCACCTCCACCTCCCTTATACAGC TCGTC-3′ (the glycine linker site is underlined) was inserted into the pGEM-T Easy vector (Promega) to produce pG-*GFP1*. The full-length *HT1* cDNAs were amplified using Pfu DNA polymerase (Stratagene) with the oligonucleotides 5′-CCATATGTCTGGTTTATGTTTCA-3′ and 5′-CCAACGCGTTGGTGTACATCAATAAAGTATCATTATA TATC-3′, and were inserted into the pGEM-T Easy vector to produce pG-*HT1*-C. The NdeI–BstXI fragment of pG-*HT1*-C was inserted into the pG-*GFP1* to produce pG-*HT1-GFP*. The NcoI-BsrGI fragment of pG-*HT1-GFP* was inserted into the NcoI/BsrGI site of pKS(+)GFP (Sugimoto *et al.*, 2007) to produce KS-35S-*HT1-GFP*. The ApaI-SmaI fragment of KS-35S*HT1-GFP* containing the CaMV 35S promoter and *HT1-GFP* translation fusion was inserted into the ApaI/SmaI site of pPZP2H-lac. Transgenic Arabidopsis plants were generated by *Agrobacterium tumefaciens*-mediated transformation.

### Confocal laser scanning microscopy

Leaf specimens were observed using a fluorescence microscope (IX70, Olympus) equipped with a spinning-disc confocal laser scanning unit (CSU10, Yokogawa) and a cooled CCD camera head system (CoolSNAP HQ2, Photometrics), as previously described (Hashimoto-Sugimoto *et al.*, 2013). To determine the location of GFP-tagged HT1, serial optical sections of whole guard cells were obtained at 1 μm intervals. To localize plasma membranes with FM4-64 staining, the leaves were treated with 33 µM FM4-64 for 10min. GFP and FM4-64 fluorescence were detected with appropriate optical settings as previously described (Hashimoto-Sugimoto *et al.*, 2013). To observe plasmolysed guard cells, leaves were mounted in 0.4M mannitol for 30min and then observed.

## Results

### Isolation and characterization of *ht1* alleles

**Fig. 1.**
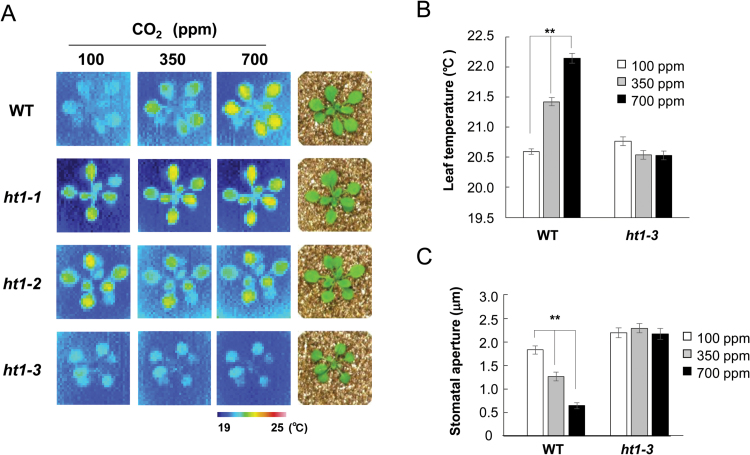
The *ht1-3* mutant has constitutively lower leaf temperatures due to a defect in the stomatal closure response to CO_2_. (A) Thermal images of WT and *ht1* mutants at different [CO_2_]. Three-week old plants were subjected to low (100ppm), normal (350ppm) and high (700ppm) [CO_2_]. WT and *ht1* allele (*ht1-1* to *ht1-3*) seedlings used for thermal images are shown on the right. (B) Leaf temperature quantified from infrared images (means ±SEM; *n*=8 leaves from four plants per treatment). (C) Response of stomatal aperture to CO_2_ in WT and *ht1-3* mutant plants. Three-week-old plants were incubated at the indicated [CO_2_]. Data are means ±SEM. (*n*=120) of three independent experiments. ** Indicates statistically significant difference as assessed by a one-way ANOVA with Tukey-Kramer multiple comparison tests (B, C; *P*<0.01).

In order to isolate the CO_2_-signaling genes, we screened for mutants with altered stomatal CO_2_ responses by monitoring leaf temperature changes using thermography, since these are indicators of changes in stomatal aperture (Hashimoto *et al.*, 2006). To date, we have isolated five *ht1* alleles using the thermal screening technique (*ht1-1*, *ht1-2*, *ht1-3*, *ht1-4*, and *ht1-5*; Fig. 1A and Supplementary Fig. S1 at *JXB* online). All five of the mutant alleles were ethyl methansulfonate (EMS)-induced mutations of an M_2_ population and were found to contain single base-pair alterations in the *HT1* gene (see below). In addition, we obtained the *ht1-6* mutant, which has a missense mutation, from the Arabidopsis TILLING project, and a T-DNA insertional mutant line, *ht1-7* (FLAG_446H04), from the INRA collection (see below and Supplementary Fig. S1). All mutants except *ht1-3* had higher leaf temperatures under low [CO_2_], and leaf temperature changes in response to CO_2_ were altered in a manner similar to that in *ht1-1* or *ht1-2* (Hashimoto *et al.*, 2006) (Fig. 1A and Supplementary Fig. S1). In contrast, *ht1-3* had constitutively lower leaf temperatures even in high [CO_2_] (Fig. 1A, B; WT, *P*=1.3×10^−12^; *ht1-3*, *P*=0.56; one-way ANOVA). Stomatal opening induces evaporative cooling of the leaf, and we found the stomata of *ht1-3* were wide open regardless of the atmospheric [CO_2_] (Fig. 1C; WT, *P*=6.1×10^−21^; *ht1-3*, *P*=0.69; one-way ANOVA). This result indicates that the *ht1-3* mutant has a CO_2_-insensitive phenotype. When the *ht1-3* plants were backcrossed to the wild type, the resultant heterologous *ht1-3* (+/–) plants had lower leaf temperatures even under high [CO_2_], thus showing a CO_2_-insensitive phenotype (Supplementary Fig. S2A, B; WT, *P*=1.56×10^−5^; *ht1-3* (+/–), *P*=0.23; *ht1-3* (–/–), *P*=0.23; Welch’s *t*-test). To confirm the genetic basis for the CO_2_ response observed in the *ht1-3* mutant, we produced transgenic WT plants carrying *HT1* genomic DNA with a heterologous *ht1-3* mutation. The transgenic T_1_ plants (WT::*HT1* ^R102K^) also had lower leaf temperatures under high [CO_2_] (Supplementary Fig. S2C, D; *P*=2.5×10^−18^; one-way ANOVA). These results showed that *ht1-3* (HT1^R102K^) is a dominant mutation.

### HT1 is a Raf-like Group C MAPKKK

**Fig. 2.**
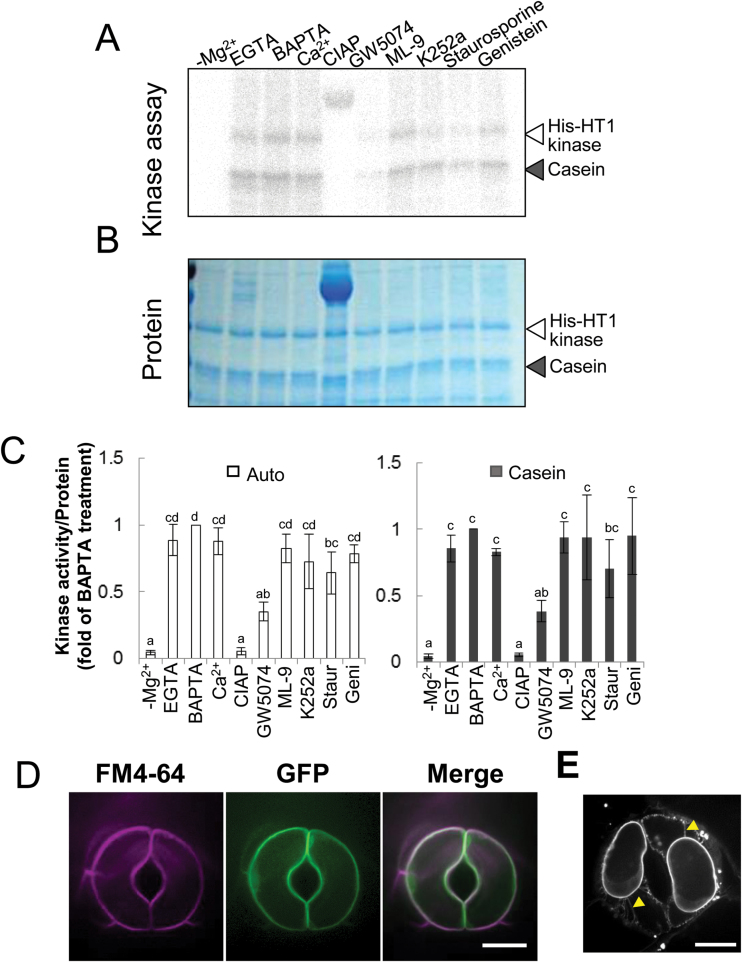
HT1 is a plasma membrane-localized Raf-like MAPKKK. (A) *In vitro* kinase assays using recombinant HT1 with calcium chelators or kinase inhibitors. The open arrowhead indicates signals from autoradiograms of autophosphorylated proteins. The solid arrowhead indicates phosphorylation of casein. EGTA and BAPTA, calcium chelators; GW5074, Raf-1 kinase inhibitor; ML-9, inhibitor of MYLK and CaMK; K252a and staurosporine, Ser/Thr kinase inhibitors; genistein, tyrosine kinase inhibitor. Calf intestinal alkaline phosphatase (CIAP) was used as a negative control. (B) Coomassie staining of the protein used in the kinase assay served as a loading control. (C) Quantified autophosphorylation (left) and casein phosphorylation (right) corrected for protein content as quantified by Coomassie staining and calculated as values relative to BAPTA-treated samples. Data are means ±SD (*n*=3). Different letters above the bars indicate statistically significant differences between the inhibitor treatments, assessed by a one-way ANOVA with Tukey-Kramer multiple comparison tests at *P*<0.05. (D) Subcellular localization of GFP-HT1. Dual observations of FM 4-64-stained plasma membranes and GFP-HT1. Optical sectional images of FM4-64 (magenta), GFP (green) and their merged images are shown. (E) Plasmolysed guard cells expressing GFP-HT1. Epidermal peels of GFP-HT1 transgenic plants were treated with 0.4M mannitol for 30min. Arrowheads indicate Hechtian strands. Scale bars indicate 10 µm (D, E).

MAPKKKs (mitogen-activated protein kinase kinase kinases), which are involved in various physiological, developmental and hormonal responses, are divided into three groups (Groups A to C) (Ichimura *et al.*, 2002). The HT1 kinase At1g62400 is, from its amino acid sequence, predicted to be a Raf-like Group C MAPKKK (Ichimura *et al.*, 2002), and few functions of Group C MAPKKKs in Arabidopsis have yet been determined. To examine the properties of HT1 kinase, we performed an *in vitro* kinase assay using calcium chelators and protein kinase inhibitors (Fig. 2A–C). We generated a His-tagged HT1 protein using *E. coli*. This recombinant protein (His-HT1) was capable of autophosphorylation and casein phosphorylation (Fig. 2A). Phosphorylation activities of HT1 were completely lost when the assay was performed in a buffer without magnesium ions (–Mg^2+^). In the absence of Mg^2+^, the phosphorylation level was almost identical to that of the negative control, in which phosphorylated proteins were dephosphorylated by CIAP (Fig. 2A–C). Calcium ions did not affect the HT1 kinase activity because the calcium chelators EGTA and the more effective BAPTA did not inhibit phosphorylation. Furthermore, these phosphorylation levels were not significantly different from those containing calcium ions (Fig. 2A–C). Few differences in the HT1 phosphorylation activity were observed in the presence of ML-9 or genistein (Fig. 2A–C). ML-9 is an inhibitor of myosin light chain kinase (MYLK) and calmodulin kinase (CaMK), and genistein is a tyrosine kinase inhibitor. K252a and staurosporine, which are broad-spectrum inhibitors of protein kinases including Ser/Thr kinase, partially decreased the HT1 phosphorylation activity (Fig. 2A–C). The most effective inhibitor of HT1 kinase activity was GW5074, a Raf-1 kinase inhibitor. The HT1 autophosphorylation activity and casein phosphorylation activity at the presence of GW5074 were, respectively, 35% and 38% compared with the BAPTA treatment (Fig. 2A–C). Taken together, these results indicate that HT1 kinase is a Ca^2+^-independent Raf-like MAPKKK with Ser/Thr kinase activity.

### Intracellular localization of HT1

To determine the subcellular localization of HT1, five independent transgenic lines of 35S::*GFP-HT1* were analysed by confocal laser scanning microscopy. GFP fluorescence was evenly distributed around the periphery of the guard cells in the 35S::*GFP-HT1* plants (Fig. 2D). We stained live leaf tissue of 35S::*GFP-HT1* plants with the lipophilic dye FM4-64, which produces a bright red fluorescence in plasma membranes just after application (Fischer-Parton *et al.*, 2000; Bolte *et al.*, 2004). Confocal microscopy analysis revealed a precise overlap of GFP-HT1 fluorescence with FM4-64 fluorescence immediately after staining (Fig. 2D). To confirm this localization, the cotyledons of the transgenic plants were plasmolysed with 0.4M mannitol for 30min. GFP fluorescence in the plasmolysed guard cells was observed in Hechtian strands, parts of the plasma membrane connected to the cell wall (Fig. 2E, arrowheads), further supporting the localization of HT1 in the plasma membranes. The secondary-structure prediction programs SOSUI (http://harrier.nagahama-i-bio.ac.jp/sosui/sosui_submit.html) (Hirokawa *et al.*, 1988) and TMHMM (v. 2.0; http://www.cbs.dtu.dk/services/TMHMM/) (Krogh *et al.*, 2001) indicated that HT1 kinase has no transmembrane domains. Overall, the results therefore suggest that HT1 associates with plasma membranes.

### *In vitro* kinase assay using HT1^R102K^

**Fig. 3.**
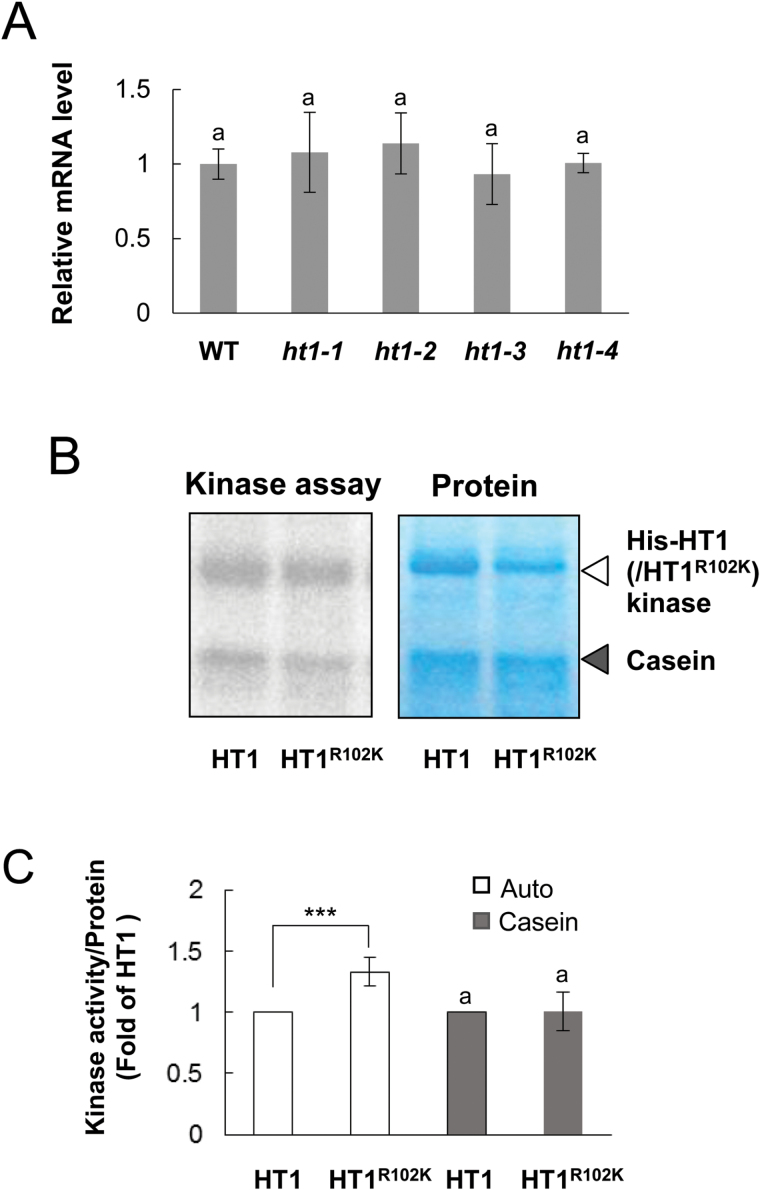
*HT1* gene expression level and kinase activity with or without the *ht1-3* mutation. (A) qRT-PCR analysis of *HT1* mRNA. Expression in aerial tissues of 3-week-old WT and mutant (*ht1-1*, *ht1-2, ht1-3* and *ht1-4*) plants. The *UBQ10* gene was used as an internal standard for cDNA amounts. Error bars on each column indicate the standard deviation from four biological replicates. The statistical significance was determined by a one-way ANOVA with Tukey-Kramer multiple comparison tests. The same letter indicates no significant difference (*P*>0.05). (B) *In vitro* kinase assays using recombinant HT1 and HT1^R102K^ with or without casein. Coomassie staining of the His-fusion protein and casein served as the loading controls. (C) Quantified autophosphorylation (Auto) and casein phosphorylation (Casein) corrected for protein content as quantified by Coomassie staining and calculated as values relative to HT1 phosphorylation activity. Error bars on each column indicate the standard deviation from seven biological replicates. ***, indicates a significant difference (*P*<0.001), from the HT1 phosphorylation levels as assessed by a paired *t*-test.

To examine whether the *ht1-3* mutation affects the expression level of the *HT1* gene, transcript levels were analysed by qRT-PCR (Fig. 3A). The *HT1* mRNA abundance in mature leaves of *ht1-3* seedlings was not significantly different from that of WT or other *ht1* mutants (*ht1-1*, *ht1-2* and *ht1-4*) (Fig. 3A)

We have previously demonstrated that site-directed mutagenesis of *ht1-1* (His-HT1^R211K^) severely reduces phosphorylation activity, and that the analogous His-construct of *ht1-2* (His-HT1^Δ136–149^), which contains a 14-amino-acid deletion, disrupts phosphorylation activity (Hashimoto *et al.*, 2006). Thus, the kinase activities of the WT HT1 and its two mutants are strongly linked to the phenotypes observed in whole plants. This result led us to expect that the *ht1-3* mutation may increase phosphorylation activity. To verify this hypothesis, we produced His-tagged recombinant HT1 protein with the *ht1-3* mutation (His-HT1^R102K^) and compared the HT1 kinase activities by *in vitro* kinase assays (Fig. 3B). The autophosphorylation activity in His-HT1^R102K^ was significantly enhanced compared with that of His-HT1 (Fig. 3C). On the other hand, the phosphorylation activity of His-HT1^R102K^ to the universal substrate casein was not significantly different from that of His-HT1 (Fig. 3C). The inhibitory effect of kinase inhibitors on His-HT1 ^R102K^ kinase activity was also similar to that of His-HT1 (see Supplementary Fig. S3).

### The *ht1-3* mutation replaces Lys with Arg at 102 – a not highly conserved region

**Fig. 4.**
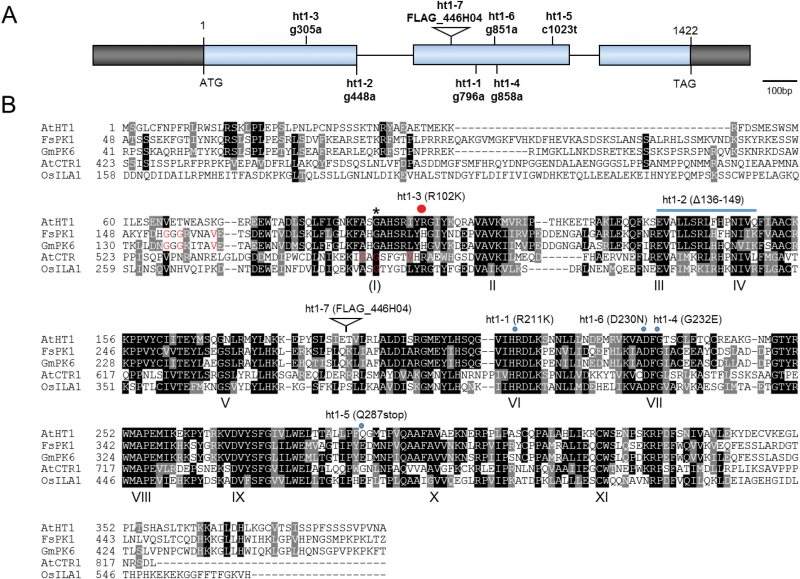
Positions of *ht1* mutation sites. (A) Structure of the *HT1* gene and positions of *ht1* mutations and T-DNA insertion sites. The sites of nucleotide substitutions found in the *ht1* alleles are indicated. Exons are denoted by blue boxes and introns by lines between the boxes. Black boxes highlight the 5′- and 3′-untranslated regions at left and right, respectively. (B) Multiple sequence alignment of HT1 and several representative Raf-like MAPKKK sequences using ClustalW. Conserved subdomains of the protein kinase family are indicated by Roman numerals (Hanks *et al.*, 1988). The consensus amino acids (Gly-X-Gly-X-X-X-X-Val) of subdomain I are not evident in AtHT1 and OsILA1, but there is an invalid Gly (indicated by an asterisk) in the potential subdomain I that is indicated as (I). Previously reported positions of subdomain I in FsPK1, GmPK6, OsILA1, and AtCTR1 are indicated with red letters. The positions of amino acid changes or the T-DNA insertion sites for the seven *ht1* alleles are indicated on the sequences of AtHT1. AtHT1, *Arabidopsis thaliana* high leaf temperature 1 (accession no. Q2MHE4); FsPK1, *Fagus sylvatica* protein kinase 1 (accession no. CAC09580); GmPK6, *Glycine max* protein kinase 6 (accession no. NP_001238530); AtCTR1, *Arabidopsis thaliana* constitutive triple response 1 (accession no. NP_195993); OsILA1, *Oryza sativa* increased leaf angle 1 (accession no. NP_001058617). The positions of amino acids changes found in the *ht1* alleles are indicated above the alignments.

We aligned and compared the amino acid sequences of several Raf-like MAPKKKs, characterized the HT1 sequences, and mapped the *ht1* mutation sites. Protein kinases have eleven conserved subdomains. One of these, subdomain I, has the consensus sequence Gly-X-Gly-X-X-(Gly)-X-Val, and the glycine-rich motif forming a loop is thought be involved in anchoring ATP (Hanks *et al.*, 1988). The consensus sequences are not clearly conserved in the HT1 protein kinase or in some other kinases, including OsILA1 (increased leaf angle *1*), a recently identified kinase of MAPKKKs in rice (Ning *et al.*, 2011) (Fig. 4B). However, there are regions similar to the consensus sequences with a highly conserved Gly that could correspond to subdomain I [(I) in Fig. 4B]. The other subdomains (II to XI) of protein kinases were highly conserved in HT1, and recombinant HT1 protein had phosphorylation activity (Hashimoto *et al.*, 2006) (Figs 2A and 4B).

We have previously reported that recessive mutations of *ht1-1* and *ht1-2* cause reduced and no phosphorylation activity, respectively (Hashimoto *et al.*, 2006). The *ht1-1* mutation causes a single amino acid substitution (R211K) at a highly conserved Arg in subdomain VI (Fig. 4). The *ht1-2* mutation results in the deletion of amino acids (*ht1-2*; Δ136–149) in the highly conserved regions of the catalytic domains corresponding to subdomains III and IV (Hashimoto *et al.*, 2006) (Fig. 4). We found that the mutations of *ht1-4* and *ht1-6* contained G to A transitions at positions 858 and 851 that are predicted to result in changes from Gly to Glu at position 232 and Asp to Asn at 230, respectively (Fig. 4). These are invariant Gly and Asp residues within subdomain VII. The *ht1-5* mutation contains a C to T transition at position 1023 that is predicted to result in a stop codon at amino acid 287 (Q287stop); thus, the mutation would lead to a truncated HT1 protein lacking subdomains X and XI (Fig. 4). The site of a T-DNA insertion in *ht1-7* (FLAG_446H04) was found to be in the second exon and is predicted to result in disruption of the *HT1* gene (Fig. 4). All these recessive mutations either convert an amino acid (*ht1-1*, *ht1-4* and *ht1-6*), lead to deletion of amino acids (*ht1-2* and *ht1-5*), or disrupt the T-DNA insertion (*ht1-7*) at highly conserved regions in the catalytic domain.

The *ht1-3* mutation was found to contain a G to A transition at position 305 that results in an Arg being replaced by Lys at amino acid 102 (R102K) (Fig. 4). The position is not highly conserved among protein kinases; for example, some plant kinases such as FsPK1 and its homologous protein GmPK6 have His at this position (Fig. 4B). Furthermore, a variety of amino acids other than Arg, including His, Asn and even Lys, can be present at this position in kinases of animals and fungi (Hanks *et al.*, 1988). Arg and Lys residues are structurally similar and are classified as basic amino acids. This result led us to wonder why this amino acid replacement caused such a drastic change in stomatal response to CO_2_.

### Structural model of the HT1 kinase

**Fig. 5.**
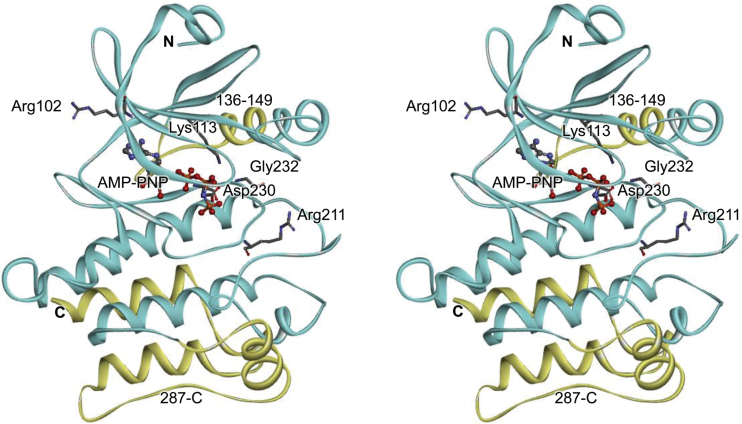
Stereo view of a structural model of HT1 kinase. The substituted and the deleted amino acid positions in the *ht1* mutants are shown as stick residues and yellow ribbons, respectively. The docked substrate analogue, AMP-PNP, is shown as a ball-and-stick model. Lys 113 is an invariant catalytic lysine in subdomain II of protein kinases that is crucial for binding to ATP.

To gain insights into the functional meaning of the *ht1-3* mutation, we constructed a 3D structural model of the HT1 kinase and examined the mutated site. The 3D structural model of 273 amino acids of the HT1 catalytic region (residues 73–345) was generated by homology modeling using Modeller (Fiser and Šali, 2003) (Fig. 5). The template used to build the HT1 kinase model was derived from the crystal structure of the CTR1 kinase domain (chain A, Protein Data Bank code 3ppz) (Mayerhofer *et al.*, 2011). Since the ligand of CTR1 in 3ppz is staurosporine, an ATP analog, AMP-PNP was docked at the ATP binding site of HT1 by superimposing the main chain structure on that of another HT1 analog, cAMP-dependent protein kinase (pdb code 4dfx) that is bound to AMP-PNP (Fig. 5). CTR1 is a negative regulator of the ethylene response pathway in Arabidopsis and is a member of the Raf-like MAPKKKs of Group B that have extended N-terminal domains (Kieber *et al.*, 1993; Ichimura *et al.*, 2002). HT1 kinase has a short N-terminal domain that is unlike that of CTR1; however, the catalytic domains of HT1 are relatively similar to those of CTR1. Notably, R102 of HT1 corresponds with the Arg present at position 567 in CTR1 (Fig. 4B).

Lys 113 is an invariant residue in subdomain II that is essential for phosphorylation activity in protein kinases; a replacement of this amino acid by Trp abolishes kinase activity (Hanks *et al.*, 1988; Hashimoto *et al.*, 2006). The highly conserved residues in subdomain VI (corresponding to R211; ht1-1) and subdomain VII (corresponding to D230; ht1-6, and G232; ht1-4) have been implicated in ATP binding (Hanks *et al.*, 1988). Consistent with these reports, four amino acids (Lys 113, R211, D230, and G232) are located close to the above-mentioned ATP analog (AMP-PNP) (Fig. 5). Deletions of the amino acids caused by the mutations *ht1-2* (Δ136–149) or *ht1-5* (Δ287–C) also are likely to destroy the kinase structure and lead to loss of function. The *ht1-1* and *ht1-2* loss-of-function mutations of HT1 reduce or disrupt HT1 kinase activity and result in constitutive stomatal closure even under low [CO_2_] (Hashimoto *et al.*, 2006). This result is consistent with the finding that all recessive *ht1* alleles cause a deletion or replacement of the highly conserved amino acids at kinase catalytic domains and result in plants with higher leaf temperatures even in low [CO_2_] (Fig. 1 and Supplementary Fig. S1).

On the other hand, the *ht1-3* mutation site (R102) is not a highly conserved residue, and the replacement of Arg102 by Lys seems not to affect the HT1 kinase structure severely. In the model of HT1 protein structure, Arg102 seems to protrude from the surface of the kinase. When the Arg is substituted with Lys, the side chain becomes shorter, and thus the positive charge stretches less outward (Fig. 5). The R102K change may therefore affect HT1 interactions with its targets.

### CO_2_ responses in the *ht1-3* and *ht1-2* mutants were completely defective

**Fig. 6.**
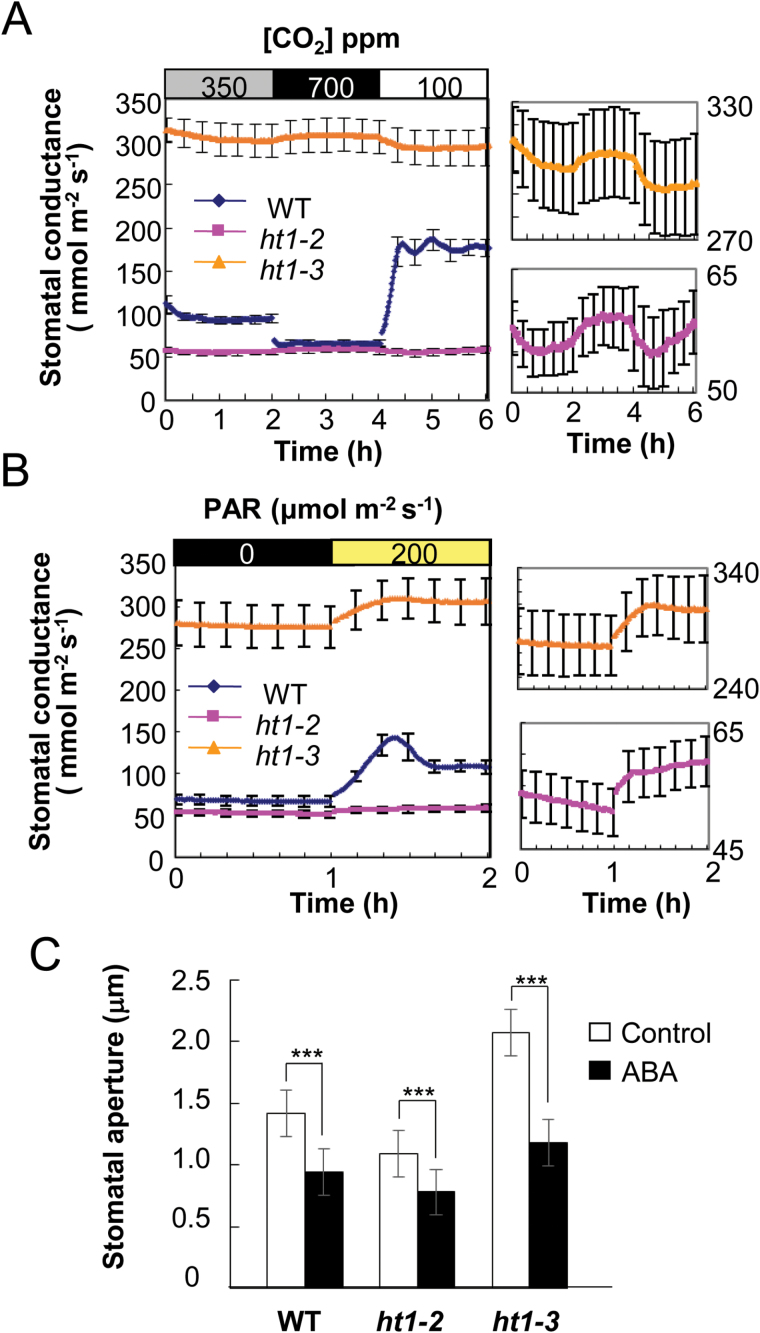
CO_2_, light and ABA responses in the *ht1-2* and *ht1-3* mutants. Stomatal conductance in response to [CO_2_] changes (A) and light (B). The graphs on the right are enlargements of sections of the graph on the left. Data represent means ±SEM (*n*=4) from 3–4-week-old plants. (C) Comparison of ABA-induced stomatal closure in WT and the *ht1* mutants. Detached leaves were incubated with or without 10 µM ABA. Data represented are the means of 120 stomatal apertures ±SEM (three experiments). ***, indicates statistically significant differences (*P*<0.001) between the control and ABA-treated plants determined with a Welch’s *t*-test.

The dominant *ht1-3* mutation leads to stomatal opening due to guard cell insensitivity to high [CO_2_] and, therefore, *ht1-3* and the other loss-of-function *ht1* alleles are likely to have opposite effects on stomatal opening or closing in response to CO_2_. To examine the nature of the *ht1-3* mutation, we compared the CO_2_, light and ABA responses of the *ht1-3* mutant with the responses of the severe loss-of-function mutant *ht1-2*. First, we investigated how stomatal conductance responded to changing [CO_2_] in the leaves of *ht1-3, ht1-2* and WT plants (Fig. 6A). In the WT, increasing [CO_2_] from 350ppm to 700ppm induced a distinct decrease in stomatal conductance, and a subsequent decrease of [CO_2_] from 700 to 100ppm induced a large increase in stomatal conductance (Fig. 6A, left). In contrast, stomatal conductance remained at a lower level in *ht1-2* and a higher level in *ht1-3*, with little change in stomatal conductance in response to CO_2_ (Fig. 6A, left). Interestingly, *ht1-3* still had an extremely small, inverse response to CO_2_, as did *ht1-2* (Fig. 6A, right)_._ These inverse responses may be the result of the activity of a counter-balancing regulator of the normal CO_2_-induced stomatal response. These observations suggest that these mutations severely disrupt stomatal CO_2_ signaling.

Next, we analysed light-induced stomatal responses in *ht1-3* mutants (Fig. 6B). Irradiation resulted in a large increase in stomatal conductance in the WT, a smaller increase in *ht1-3*, and a much smaller increase in *ht1-2* (Fig. 6B, left). In contrast to their inverse responses to changing [CO_2_], the *ht1-2* and *ht1-3* mutants did respond to changes in light intensity in the same direction as the WT responses (Fig. 6B, right).

The *ht1-3* mutant did not have the wilted phenotype under normal conditions; however, when grown without water for a week, the mutant started to exhibit a wilted phenotype compared with the WT (see Supplementary Fig. S4). As drought stress triggered a wilted phenotype in the *ht1-3* plants, it was possible that the mutant plants might be insensitive to ABA. Production of the phytohormone ABA is triggered by desiccation and induces stomatal closure, and ABA-insensitive mutants have a wilted phenotype under dry conditions. We found that the degree of stomatal closure induced by ABA in the *ht1-2* and *ht1-3* mutants was similar that of the WT (Fig. 6C; WT, *ht1-2* or *ht1-3*; *P*<0.01, for controls vs. ABA; Welch’s *t*-test), suggesting that ABA responses in these mutants are normal. This finding indicates that the wilted phenotype observed in *ht1-3* was due to insensitivity to CO_2_ but not to ABA.

## Discussion

### HT1 is a Group C Raf-like MAPKKK essential for the stomatal CO_2_ response

We performed phosphorylation assays with several kinase inhibitors and confirmed that HT1 is a Group C Raf-like MAPKKK. Almost all of the MAPKKKs that have been functionally characterized are members of either Group A or B. In contrast, most Group C members have been described only from genomic sequence analyses (Ichimura *et al.*, 2002). FsPK1, an ABA-induced protein kinase, is classified in subgroup C5, the same group as HT1. FsPK1 has Ca^2+^-dependent protein kinase activity that is inhibited by the kinase inhibitors staurosporine and genistein, indicating dual activities (Ser/Thr and Tyr protein kinases) (Lorenzo *et al.*, 2003). OsILA1, also a member of Group C, is a key factor for regulating mechanical tissue formation at the leaf lamina joint; this protein has Ser/Thr kinase activity but no Tyr kinase activity (Ning *et al.*, 2011). An *in vitro* kinase assay revealed that HT1 could be a Raf-related MAPKKK with Ca^2+^-independent Ser/Thr kinase activity (Fig. 2A). These findings demonstrate that the features of this protein kinase and others vary among the subgroups of Group C MAPKKKs. HT1 has phosphorylation activity, and mutations inducing changes in the highly conserved amino acids in the catalytic domain impair its activity (Hashimoto *et al.*, 2006). All six *ht1* recessive mutation sites were expected to alter the highly conserved amino acid residues that play a critical role in phosphorylation activity, and resulted in loss-of-function phenotypes (Figs 1, 4 and Supplementary Fig. S1). These results indicate that the kinase activity of Raf-like MAPKKK HT1 is important for stomatal CO_2_ responses.

### HT1 is localized on plasma membranes

OST1 acts as an ABA-activated SnRK2-type protein kinase (Mustilli *et al.*, 2002), and phosphorylates and activates the S-type anion channel SLAC1 (Geiger *et al.*, 2009; Lee *et al.*, 2009). OST1 and SLAC1 have also been reported to be involved in elevated [CO_2_]-induced stomatal closure (Negi *et al.*, 2008; Vahisalu *et al.*, 2008; Xue *et al.*, 2011). A recent study has indicated that a MATE-type transporter, RHC1, could interact with HT1 and overcome HT1 inhibition of downstream SLAC1 activation by OST1 under high-bicarbonate conditions (Tian *et al.*, 2015). The subcellular localization of RHC1 was reported to be the plasma membranes, whereas OST1 localizes to nuclei and the cytosol (Fujita *et al.*, 2009; Tian *et al.*, 2015). We showed that HT1 is associated with plasma membranes, indicating that it could transduce CO_2_ signals from plasma membrane-resident RHC1 to OST1 in the cytosol.

### HT1 is a master regulator in the stomatal CO_2_ response

Similar to recessive loss-of-function *ht1-2* mutants, the *ht1-3* plants had defects in their CO_2_ response; however, the *ht1-3* plants showed a functional stomatal response to light and a normal response to ABA, indicating a CO_2_-specific role for HT1 (Fig. 6) (Hashimoto *et al.*, 2006). CO_2_-induced stomatal conductance changes in *ht1-2* and *ht1-3* plants were disrupted, and the stomatal conductance in *ht1-2* and *ht1-3* remained low and high, respectively (Fig. 6A). Interestingly, we found slightly inverse responses to CO_2_ in both alleles (Fig. 6A). No other mutants have been reported that show such severe and specific damage in their CO_2_ responses. A crucial role of the HT1 kinase in CO_2_ signaling is also supported by the observation that many of the CO_2_-signaling mutants we have been isolating by thermal imaging screens are HT1 mutant alleles.

### HT1 is partially involved in the light-signaling pathway

The HT1 loss-of-function and gain-of-function mutations brought about a reduced response to light (Fig. 6). This result indicates that the functions of HT1 partially share the light-signaling pathway. Red light induces stomatal opening by reducing the intracellular [CO_2_] caused by photosynthesis (Roelfsema *et al.*, 2002, 2006); however, other research has reported that red light can induce stomatal opening when the intracellular [CO_2_] was constantly maintained (Messigger *et al*., 2006; Lawson *et al.*, 2008). A recent study has reported that the *ht1-2* mutant was impaired in red light-induced stomatal opening (Matrosova *et al.*, 2015). In our study, light induced a rise in stomatal conductance in *ht1-3* that was larger than that of *ht1-2*, although both mutants completely lost their stomatal response to CO_2_ (Fig. 6B). Therefore, analysis of the red light-induced stomatal opening response in *ht1-3* should provide more clear information about the contribution of HT1 to the red light signaling pathway and the roles of the reduced [*C*_i_]-dependent and the [*C*_i_]-independent pathways.

### *ht1-3* is a dominant mutation leading to enhanced autophosphorylation activity

The abundance of *HT1* mRNA in *ht1-3* plants was not significantly different from that in the wild type (Fig. 3A), suggesting the dominant mutation may affect post-transcriptional regulation. In the HT1 protein structure model, Arg102 seems to protrude from the surface of the kinase (Fig. 5). Arg and Lys have similar positively charged residues, but Lys has a shorter side chain and thus the positive charge would not stretch outward as much (Fig. 5). This explanation suggests that the *ht1-3* mutation might affect the kinase’s interaction with its targets. It may be hypothesized that the *ht1-3* mutation influences kinase activity because Arg102 may be located close to the kinase active site (Fig. 5); however, the kinase assays revealed that the HT1^R102K^ was not affected in its ability to phosphorylate casein (a universal substrate for a wide range of kinases) *in vitro* (Fig. 3C). This result indicates that the dominant mutation does not enhance kinase activity by itself. In contrast, HT1 autophosphorylation activity was significantly increased by the *ht1-3* mutation (Fig. 3C). This result suggests that the *ht1-3* mutation enhances the formation of HT1 oligomers and/or the efficiency of self-phosphorylation, and then the activated HT1^R102K^ kinase can interact and phosphorylate target proteins. Future studies searching for direct HT1 targets and using phosphorylation profiling by means of activated HT1^R102K^ kinase may allow further isolation of the CO_2_ signaling factors, and thus result in more detailed elucidation of the CO_2_ signaling pathways.

## Supplementary data

Supplementary data are available at *JXB* on line.

Figure S1. Thermal images of *ht1-4*, *ht1-5*, *ht1-6*, and *ht1-7* at different [CO_2_].

Figure S2. Data indicating that *ht1-3* is a dominant allele of *HT1.*

Figure S3. Inhibitory effects of kinase inhibitors on HT1^R102K^ kinases.

Figure S4. Images showing that *ht1-3* mutant plants have a wilted phenotype under mild drought stress.

Supplementary Data
