# Supplementary material for: Dominant and recessive mutations in the Raf-like kinase HT1 gene completely disrupt stomatal responses to CO2 in Arabidopsis
Source: J Exp Bot. 2016 Mar 31;67(11):3251–61. doi: 10.1093/jxb/erw134 (PMC4892718; doi:10.1093/jxb/erw134)
Supplement: Supplementary Data [file supp_67_11_3251__index.html]

Dominant and recessive mutations in the Raf-like kinase HT1 gene completely disrupt stomatal responses to CO2 in Arabidopsis — Dominant and recessive mutations in the Raf-like kinase HT1 gene completely disrupt stomatal responses to CO2 in Arabidopsis — Supplementary Data 

# Dominant and recessive mutations in the Raf-like kinase *HT1* gene completely disrupt stomatal responses to CO2 in Arabidopsis

## Supplementary Data

Data files

- supplementary\_figures\_S1\_S4.pdf - Supplementary Data
