# Supplementary material for: Dominant and recessive mutations in the Raf-like kinase HT1 gene completely disrupt stomatal responses to CO2 in Arabidopsis
Source: J Exp Bot. 2016 Mar 31;67(11):3251–61. doi: 10.1093/jxb/erw134 (PMC4892718; doi:10.1093/jxb/erw134)
Supplement: Supplementary Data [file supp_erw134_supplementary_figures_S1_S4.pdf]

Supplementary Data

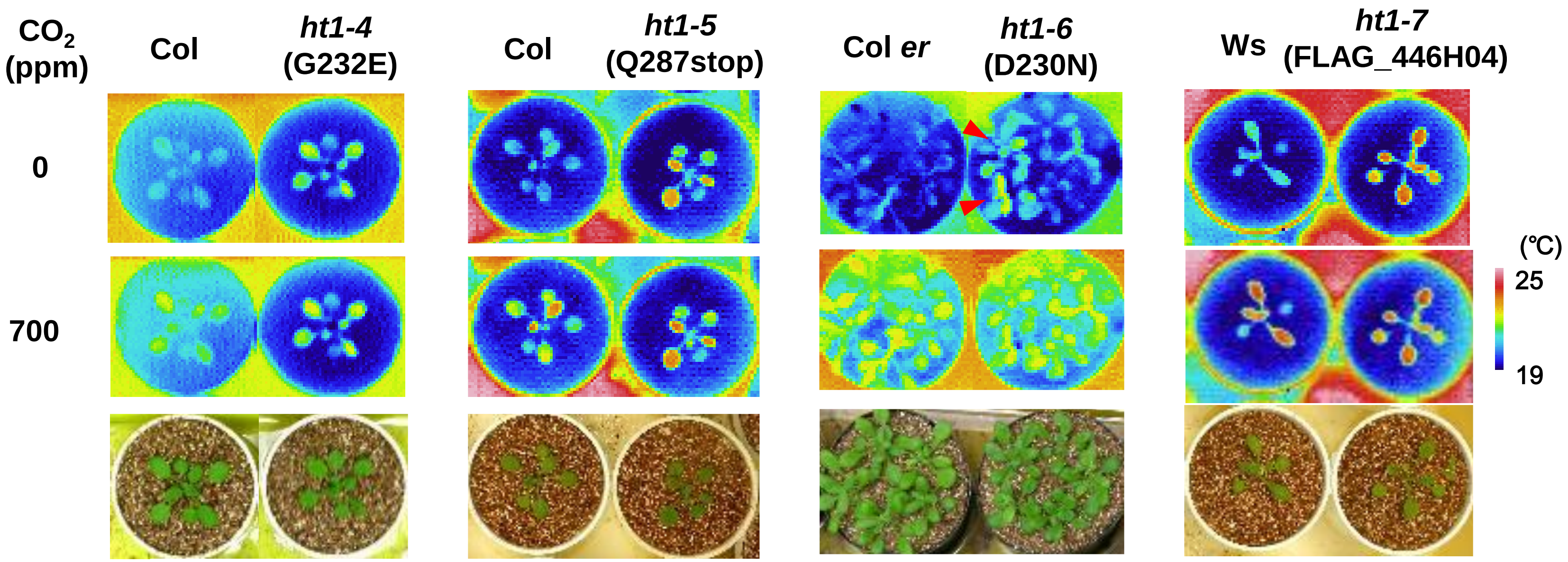

Fig. S1. Thermal images of *ht1-4*, *ht1-5*, *ht1-6* and *ht1-7* at different [CO_2].

Leaf temperature of *ht1-4* (G232E), *ht1-5* (Q287stop), *ht1-6* (D230N) and *ht1-7* (FLAG_446H04) and WT plants (Col, Col *er* or Ws). Plants were subjected to low (0 ppm) and high (700 ppm) [CO_2]. *HT1* tilling line plants with the homozygous mutation of *ht1-6* (D230N) are indicated by red triangles. All recessive *ht1* mutants showed higher leaf temperatures under low [CO_2].

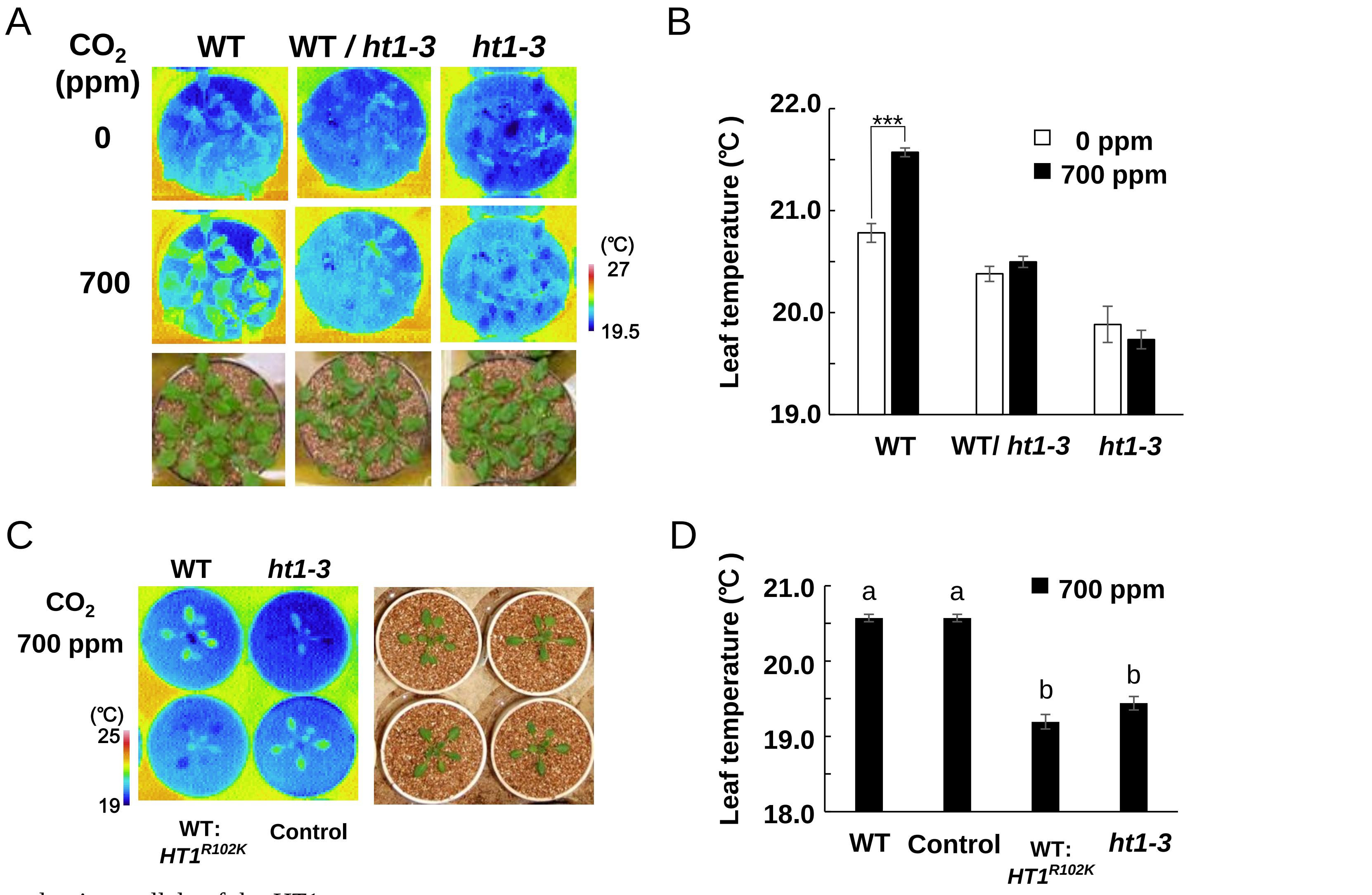

Fig. S2. *ht1-3* is a dominant allele of the *HT1*.

(A) Thermal images of heterozygous WT/*ht1-3* seedlings under low and high [CO_2]. (B) Leaf temperature calculated from the quantification of infrared images (means ± SEM; *n* = 8 leaves of the four plants per condition). The statistical significance was determined by one-way ANOVA with Tukey-Kramer multiple comparison tests (***, $P < 0.001$). (C) Thermal image of the transgenic plant carrying the HT1 gene with the dominant *ht1-3* mutation. WT: *HT1*^{R102K}, Transgenic T1 seedling harboring transgenes with the *HT1* genomic sequence containing the *ht1-3* mutation. The control was a transgenic plant harboring the vector pBI101. (D) Leaf temperature calculated from the quantification of infrared images (means ± SEM; *n* = 10 leaves of the four plants). Different letters above bars indicate statistically differences between the lines ($P < 0.01$).

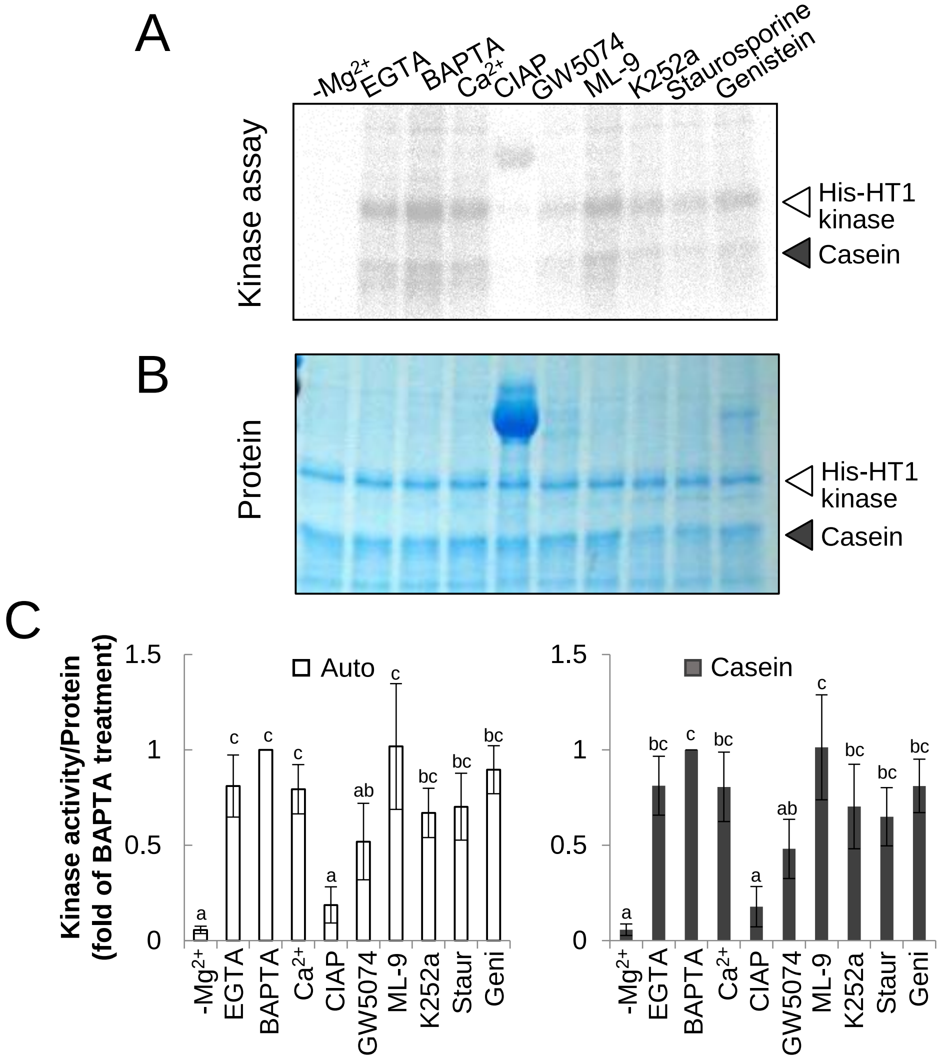

Fig. S3. Inhibitory effects of kinase inhibitors on HT1^{R102K} kinases.
(A) *In vitro* kinase assays using recombinant HT1^{R102K} with calcium chelators or kinase inhibitors. The open arrowhead indicates signals from autoradiograms of autophosphorylated proteins. The solid arrowhead indicates phosphorylation of casein. (B) Coomassie staining of the protein using kinase assay served as a loading control. (C) Quantified autophosphorylation (left) and casein phosphorylation (right) corrected for protein as quantified by Coomassie staining, and calculated as values relative to BAPTA treated ones. Data are means ± SD. (n = 3). Different letters above the bars indicate statistically significant differences between the inhibitor treatments, assessed by a one-way ANOVA with Tukey-Kramer multiple comparison tests at $P < 0.05$.

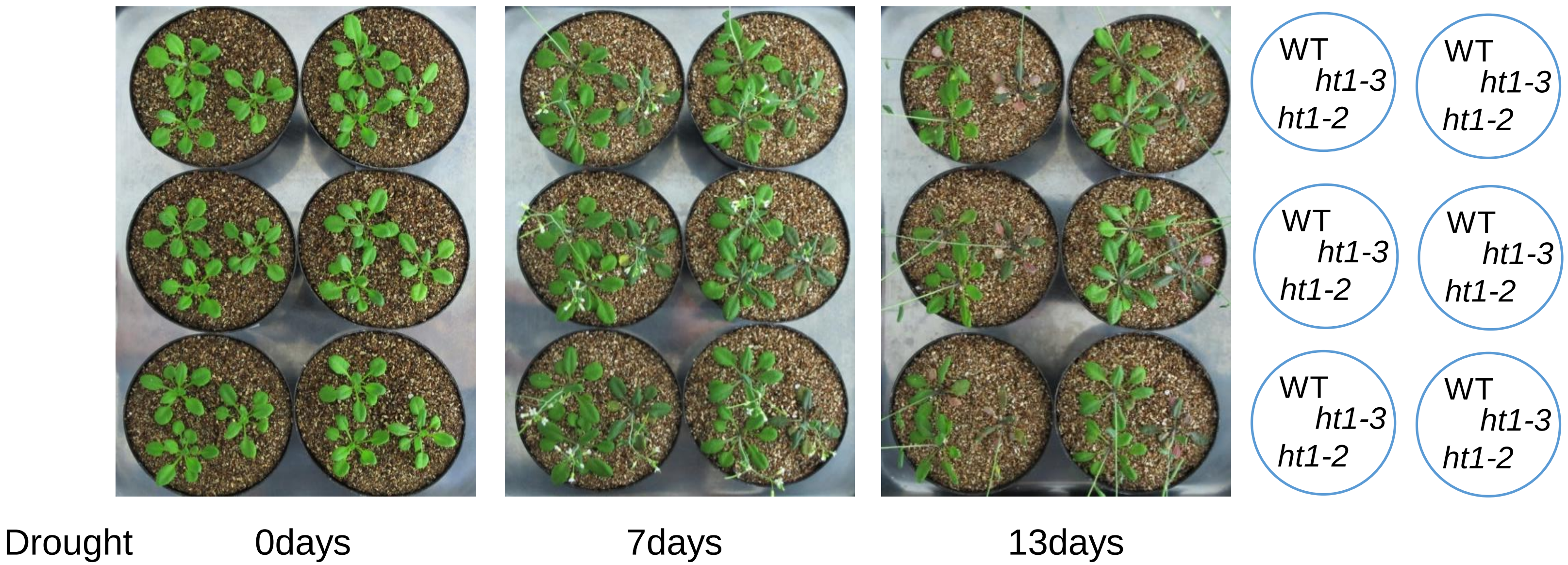

Fig. S4. *ht1-3* mutant plants had a wilted phenotype with mild drought stress.

Twenty-five-day-old plants were subjected to drought stress by water being withheld for 7 or 13 days.
